# Are early readers sensitive to the patterns in bisyllabic english words?

**DOI:** 10.1007/s11881-026-00371-4

**Published:** 2026-07-16

**Authors:** Devin M. Kearns, Christina Novelli

**Affiliations:** 1 North Carolina State University, Raleigh, NC USA; 2 University of Georgia, Athens, GA USA

**Keywords:** Word recognition, Decoding, Reading, Syllables

## Abstract

Elementary students develop sensitivity to English sound-spelling patterns through instruction and practice. However, it is unclear whether this extends to subtle patterns and whether sensitivity varies by age or reading skill. One such pattern involves the pronunciation of a single-letter vowel (V) in the first syllable of bisyllabic words. A vowel letter is more likely to have a long (tense) sound when followed by a single consonant letter (C) and vowel letter (VCV, e.g., *labor*) than a short (lax) one (Kearns, 2020). However, short-vowel pronunciations (e.g., *habit*) are common). This study examines whether early readers exhibit sensitivity to this long-vowel tendency and whether this varies by grade or reading skill. Grade 2 (*n* = 18), Grade 3 (*n* = 28), and Grade 4 (*n* = 29) students with and without reading difficulties (RD) read 48 five-letter bisyllabic CVCVC pseudowords (e.g., *losal*). First-vowel pronunciations were recorded. Long-vowel pronunciation frequencies were compared across grades and with long-vowel frequencies in a lexical database (Zeno et al., 1995). Students used long vowel pronunciations less frequently than expected based on the frequency of polysyllabic words in the database, pronouncing them at a rate closer to that found in monosyllabic words. Grade 3 and 4 students used long vowel pronunciations more often than younger students and those with RD. Long vowel usage was notably higher for the letter *O* than other vowels, consistent with the more frequent occurrence of long pronunciations for that letter. Students show limited sensitivity to the subtle VCV long-vowel pattern, indicating that while acquiring this knowledge, it is not quickly consolidated, possibly due to variability in the pattern or differing learning rates.

English is a quasiregular orthography that requires readers to navigate letter-sound inconsistencies and predict pronunciations for unfamiliar words using multiple subtle and often unreliable cues; this presents a challenge for the reader but an opportunity to test hypotheses about the learning mechanism in the acquisition of word-pronunciation skill, particularly whether readers increase their skill via statistical learning. Reading is parasitic on speech, so the orthography itself encodes the lexical statistics in the phonological lexicon. For example, in the spoken English lexicon, short (lax) vowel pronunciations occur more frequently than long (tense) ones although the same six letters, *A*,* E*,* I*,* O*,* U*, and *Y*, can represent either sound in the written lexicon (see examples given in Table 1,^1^), especially in polysyllabic words. This presents a challenge when reading unfamiliar words (that is, words never seen or heard before). Imagine reading about *Tucson* for the first time: Should the first vowel letter be pronounced with the short sound or the long (*tuck-sun* is a plausible pronunciation of *Tucson*)? This paper addresses the pronunciations early readers use when encountering words with these letters and the cognitive mechanisms that may explain their behavior.

**Table 1:** Pronunciation of first-syllable vowels (V_1_) in VCV polysyllabic words

V_1_ Pronunciation	A	E	I	O	U
Short	*habit*	*select*	*linen*	*topic*	*upend*
Long	*baby*	*remote*	*pilot*	*open*	*music*
Other*	*water*	*secure*	*kilo*	*cocoon*	*guru*

*Not a complete list of possibilities; for illustration only

English orthography contains some clues to help the reader use the intended pronunciation—especially the first single-letter vowel in a polysyllabic word (e.g., the *A* in *later*,* basket*, and *rabbit*): A salient feature is the number of consonant letters between the vowels. If the first vowel letter precedes two consonant letters, the vowel sound is short (V_1_ in V_1_CCV_2_^2^ as the *A* in *basket* or *rabbit*).^3^This feature is mostly reliable in bisyllabic words (Kearns, 2020, but see. /ə/ for* O* in *collect*, /ɑ/ for *A* in *water* or /ɔ/ in *falter*). If the vowel letter precedes only one consonant letter, the reader cannot easily predict the V_1_ pronunciation. For example, the sound of *I* differs for *viper* [/aɪ/], *linen* [/ɪ/], *machinery* [/i:/], and *flexible* [/ə/] (see Table 1 for examples).

However, the pronunciation of the first vowel in V_1_CV_2_ words is not arbitrary either: In bisyllabic V_1_CV_2_ words, the first vowel letter often has a long sound, although there are also many instances of short or reduced vowel pronunciations. Examining the degree to which readers pronounce^4^ V_1_ in printed polysyllabic with a long or short sound provides an opportunity to examine how readers process words containing patterns that are complex (pronunciation selection requires processing across two syllables and at least three letters) and subtle (involving small differences in the frequencies of orthographic and phonological patterns in the lexicon).

A parallel distributed processing (connectionist) account of word reading (e.g., the *triangle model*; Seidenberg & McClelland, 1989) is well-suited to explaining the factors that would affect which pronunciation is chosen (Plaut et al. 1996). Within the triangle model, the reader links orthographic input to phonological and semantic output through a network that encompasses all the knowledge the learner has acquired about the associations between letters, sounds, and meaning features, analogous to the biological process of learning by connecting neurons via axons. In simulations, these connections are represented by hidden units that mediate the relationship between orthography and phonology. The simulated learner derives pronunciations of a printed word using the information encoded in the strength of the connections between input letters, these mediating hidden units, and output pronunciations. Learning occurs when the reader adjusts the strength of the connections between the units to reduce the error when reading a subsequent word. An essential property of this system is that learning does not include direct acquisition of rule-like behavior (e.g., knowing that* m* makes the /m/ sound). The learning process is gradual and distributed across the entire network of connections.

This way of explaining word reading differs from a rule-based account (e.g., Coltheart et al., 2001) in which words are read by either applying a set of letter-sound rules (a non-lexical route) or directly accessing the word as a whole (a lexical route). The variability in vowel letter pronunciations in polysyllabic words illustrates that a set of rules to explain whether the sound is a long or short sound would require a large set of context-dependent rules—or might be impossible. Rastle and Coltheart (2000) illustrated this problem by creating a set of context-dependent rules to permit correct stress placement for polysyllabic words: Many rules were needed and, even after applying them, idiosyncrasies remained. Creating a set of rules to determine the pronunciation of V_1_ in V_1_CV_2_ would be even more complex. A connectionist model easily explains how this variability would affect a reader: The pronunciation for a given word has been shaped by the gradual change in the cognitive system resulting from experience reading words.

The connectionist account also provides an explanation of variability in reading acquisition. Readers will vary in the degree to which they acquire sensitivity to these consistencies. Younger learners and those with reading difficulty are likely to show less sensitivity to V_1_ pronunciation patterns because they have had less exposure to words with these patterns than older readers and those with typical achievement, because—due to age or learning difficulty—they learn patterns more slowly, or both. To explore these ideas, we tested whether early readers in Grades 2, 3, or 4 with or without reading difficulty (RD) read aloud a set of VCV pseudowords using long- or short-vowel pronunciations for V_1_ We (a) compared the frequency of students’ long-vowel pronunciations to the frequency of long-vowel pronunciations for those letters in a large lexical database and (b) examined whether the frequency of long-vowel pronunciations varied by grade or RD status.

## Empirical data on readers’ pronunciation sensitivities

To date, no directly relevant behavioral studies have examined whether early readers show sensitivity to the tendency of vowel pronunciation in VCV patterns. However, data from studies of adult word pronunciation provide evidence that experienced readers develop this sensitivity. Waese (2006) asked university students to pronounce bisyllabic nonwords containing VCV and VCCV patterns. They observed more long-vowel pronunciations for VCV patterns than VCCV patterns, although participants used long-vowel pronunciations less than expected. The authors suggested that this may have resulted from difficulty controlling multiple dimensions in the stimuli. Treiman and Kessler (2023) controlled for characteristics of bisyllabic words that may influence pronunciation, as Berg (2016) suggested, and observed that experienced readers demonstrated differences in pronunciation of V_1_ as a function of surrounding context. Specifically, they observed that the pronunciations of experienced readers were influenced by the vowel letter, the sonority of the following consonant, and end rimes. These observations are reflective of the frequencies found in a large corpus of English words, thereby providing evidence that experienced readers develop sensitivity to patterns from experience with words. In this study, we sought to determine if early readers also develop these sensitivities.

### Instructional relevance of understanding syllable processing

These data are relevant in the educational context. Many instructional programs, especially for students with reading difficulty, include instruction on a syllable division strategy for determining the first vowel pronunciation of a VCCV or VCV word (see Kearns et al., 2022, for a summary). Despite the popularity of syllable division strategies, there are few empirical tests concerning the value of their use. Moreover, the statistical patterns in the language seem inadequately consistent to justify their instruction (Kearns,  2020). The present study is not instructional, but it involves children’s pronunciations of vowels in polysyllabic words and provides an opportunity to consider the extent to which children use the subtle patterns targeted when teaching syllable division rules.

### Individual differences in vowel pronunciation sensitivity

Students should differ in terms of their sensitivity to the pronunciation of V_1_. We would expect that younger readers and those with RD use long-vowel pronunciations less frequently than their older peers with typical achievement. Younger and less-skilled readers have less exposure to text in general and thus fewer opportunities to read polysyllabic words with long-vowel pronunciations. This study provides evidence whether readers learn subtle tendencies in the language during the early stages of reading acquisition and whether learning varies by age or reading skill. It provides an opportunity to address the question whether learners acquire reading skill via parallel distributed processing, as suggested by Powell et al. (2006).

## The present study

Elementary-aged readers were asked to read pseudowords with a V_1_CV_2_ orthographic pattern (e.g., *telit*). Pronunciations for V_1_were analyzed to calculate the frequency with which students pronounced them with the long-vowel sound. We compared the frequency of these pronunciations to the frequency distributions found in subsets of words in a lexical database (Zeno 1995) to determine if students were sensitive to the probabilistic patterns found in words. The subsets from the database comprised only monosyllabic, bisyllabic, or polysyllabic words, each used for a different distribution comparison. The extent to which the readers’ long-vowel pronunciation frequencies aligned with the long-vowel frequency in a given subset would reflect whether readers showed sensitivity to the tendency vis-à-vis that category of words. For example, if the reader gave long-vowel pronunciations in this study with the same frequency as these pronunciations occurred in monosyllabic words, it would suggest that readers gave pronunciations based on their knowledge of vowels in monosyllabic words. In taking this approach, we follow Waese (2006) who observed that readers may rely on vowel-consonant co-occurrence patterns common in monosyllabic words.

Overall, we aimed to answer two primary research questions. The first question (RQ1) was whether developing readers show sensitivity to the distribution of long and short vowel pronunciations in printed V_1_CV_2_ words in a lexical database based on the frequency of long-vowel V_1_ pronunciations in five-letter CVCVC pseudowords. RQ1A addressed whether students gave long-vowel pronunciations with the frequency expected based on the frequency of these patterns in subsets of the Zeno et al. (1995) lexical database. We predicted that the reader pronunciation frequencies would align with the long-vowel pronunciation frequencies in the bisyllabic word subset of the database because the target pseudowords shared more structural orthographic features with bisyllabic words than any other subset of the words in the database.

RQ1B addressed whether student pronunciations were based on the predicted pronunciation of the vowel and the subsequent letter (e.g., *EL* =/il/in *telit*), addressing the possibility of influences from multi-letter dependencies (Treiman et al., 2023). We hypothesized that readers might be more sensitive to the distribution based on the vowel and the trailing consonant than the vowel alone. To address RQ1B, we examined obstruent and sonorant consonants separately because sonorant consonants are vowels in resonance or vibration quality and, therefore, their presence increases the likelihood of a short-vowel pronunciation for V1 in alignment with the short-vowel distributions in monosyllabic words.

The second question (RQ2) was whether the sensitivity varied by student characteristics or pseudoword features, specifically by (RQ2A) grade and RD status and (RQ2B) the V_1_ vowel. For RQ2A, we hypothesized that older students and students without RD would show greater sensitivity to the statistical properties of the language, such that their responses would more likely follow the frequency of long-vowel pronunciations found in the corpus. For RQ2B, we expected that more long-vowel pronunciations would be given for words with *O* in the V_1_ position, given the large number of monosyllabic and polysyllabic words with a long pronunciation of *O*—regardless of vowel-consonant pattern (e.g., in monosyllabic words like *most* and polysyllabic VCCV words like *golden*).

## Method

### Participants

Seventy-five students in Grades 2, 3, and 4 from five schools across the northeastern United States participated (Table 2). Students were screened using the *Test of Word Reading Efficiency*,* Second Edition* (Torgesen et al., 2012). A composite standard score was calculated by combining the scale scores of the two subtests and used as an indicator of reading performance (see Hall et al., 2023). Students were identified as (a) *students with reading disability* (RD) based on a composite score below 180 (< 25th %ile), (b) *students with typical achievement*,* chronologically age-matched* (TACA), defined as being within 6 months of the mean age of the students with RD, but without having difficulty (composite score above 189 [> 35th %ile]), or (c) *students with* t*ypical achievement*,* reading age-matched* (TARA) based on having the same level of performance as the students with RD based on the composite score (< 180) but being above the 35th %ile. The RD (*n* = 15) and TACA (*n* = 42) groups included students in Grades 3 and 4, while the TARA group (*n* = 18) were Grade 2 students.

**Table 2:** Demographics (*N* = 75)

Variable	Count	%
Grade		
2	18	24.0
3	28	37.3
4	29	39.4
Reading Difficulty	15	20.0
Sex		
Female	41	55.7
Male	34	45.3
English Language Learner (ELL)	7	9.3
Individualized Education Plan (IEP) Status		
With an IEP	11	14.7
Referred to Special Education	3	04.0
Retention (Repeating a Grade) Status		
Never Retained	73	97.3
Previously Retained	2	02.7
Free or Reduced-Price Lunch Eligibility		
Not Eligible	65	86.7
Eligible	7	09.3
Not Available or Reported	3	04.0
Race		
African American	5	06.7
Asian/Indian-American	7	09.3
Latino/Hispanic	1	01.3
Multiethnic	4	05.3
Other	2	02.7
White	56	74.7

### Stimuli

Pseudowords (*N* = 48) were created with a five-letter CV_1_CV_2_C structure. V_1_ was *A*,* E*,* I*, or *O*. We did not use *U* and *Y* words because they are much lower in frequency and contain variable vowel pronunciations; *U* is either /ju/ or/ uː/; *Y* is either /aɪ/ or /iː/. Items had four possible endings (*–al*,* –ic*,* –on*,* –el*) selected because words with these endings tend to have trochaic (first syllable) stress and are not strictly inflected forms. This increased the probability that the first vowel would have a stressed pronunciation (Arciuli et al., 2010). In other words, the vowel pronunciation would likely be short or long but not reduced). Filler words with different initial consonants and endings were included to make patterns less apparent.

### Procedure

The Institutional Review Board at the data-collection site approved conducting the experiment within a larger study. Words were presented, in a random order, individually on a computer screen, and students read each word aloud. All pronunciations were recorded, and the V_1_ pronunciations were scored as long, short, or other. A linguistic expert checked 10% of scores for accuracy. Interrater reliability between examiners' initial scores and the expert scores was 97% within this 10% subset, so examiners scored the remainder themselves.

### Data analyses

For RQ1, χ² goodness-of-fit tests were conducted to explore whether student subgroups used long-vowel pronunciations for each vowel with the same frequency as they occur in subsets of the lexical database. The database comprised words found in elementary texts (summed Grade 1 to Grade 5 word frequencies reported by (Zeno, 1995). We compared long-short pronunciations for different Zeno et al. subsets (RQ1A) overall and by sonority. Table 3 shows the subsets used and procedure used to calculate the subset V1 frequencies. We then examined whether the difference varied depending on the letter following V_1_ (e.g., *an* in *lanid*; Table 4; RQ1B).

**Table 3:** Categories for a calculations in analysis 1

Category	Short Name	Word Features	Pattern	Example(s)	Non-Example(s)
V_Only_ in Monosyllabic Words	Monosyll. V_Only_	1	VC	*mat*	*amid* – polysyllabic *major* – polysyllabic *magazine* – trisyllabic
V_1_ in V_1_CV_2_ Bisyllabic Words	Bisyll. V_1_CV_2_	2	VCV	*amid* *major*	*mapping* – V_1_CCV_2_ *mat –* V_only_ *menacing* –V_1_ is not *A;* trisyllabic
V_a_ in V_a_CV_b_ Polysyllabic Words	Polysyll. V_a_CV_b_	2 or more	VCV	*amid* *magazine* *major*	*mapping* – V_1_CCV_2_ *mat* – V_only_ *menacing* – V_1_ is not *A*
V_a_ in Monosyllabic Words and V_a_CV_b_ in Polysyllabic All Words	V_Only_ and V_a_CV_b_	2 or more	VCV	*mat* *amid* *major* *magazine* (twice) *menacing*	*mapping* – V_1_CCV_2_

*Note*: These analyses do not include any cases where the vowel letter is part of a digraph (e.g., main)

**Table 4:** Percentage of each pronunciation for words with single-letter vowels

Categories	A		E		I		O	
	Obstruent	Sonorant	Obstruent	Sonorant	Obstruent	Sonorant	Obstruent	Sonorant
Monosyll V_Only_								
*n*	211	171	117	104	176	163	150	80
Long	–		–	–	–	13.50	7.33	38.75
Other	3.79	22.81	–	–	0.57	–	0.67	21.25
Reduced	–	–	–	–	–	–	–	–
Short	96.21	77.19	100	100	99.43	86.50	92.00	40.00
Long (vs. Short)	–	–	–	–	–	13.50	07.38	49.21
Bisyll V_1_CV_2_								
*n*	685	102	501	91	443	73	423	94
Long	26.57	17.65	10.18	18.68	24.15	65.75	29.79	41.49
Other	6.86	11.76	0.20	1.10	2.26	5.48	9.46	17.02
Reduced	15.62	30.39	38.92	59.34	0.68	1.37	10.87	10.64
Short	50.95	40.20	50.70	20.88	72.91	27.40	49.88	30.85
Long (vs. Short)	34.28	30.51	16.72	47.22	24.88	70.59	37.39	57.35
Polysyll V_a_CV_b_								
*n*	1,693	395	1,364	439	1,371	404	1,029	430
Long	22.27	7.85	9.60	8.43	15.75	15.84	26.53	17.44
Other	8.56	7.59	0.07	0.46	2.19	5.20	7.87	6.98
Reduced	30.60	46.33	46.19	56.49	28.01	46.78	28.96	44.42
Short	38.57	38.23	44.13	34.62	54.05	32.18	36.64	31.16
Long (vs. Short)	36.60	17.04	17.87	19.58	22.56	32.99	42.00	35.88
V_Only_ and V_a_CV_b_								
*n*	2,245	430	2,633	447	1,507	418	1,169	442
Long	16.88	8.14	4.98	8.50	14.86	15.55	23.87	17.65
Other	6.95	7.91	0.04	0.67	2.12	5.50	6.93	6.79
Reduced	47.22	45.58	73.72	56.15	33.64	46.65	36.27	44.57
Short	28.95	38.37	21.27	34.68	49.37	32.30	32.93	31.00
Long (vs. Short)	36.83	17.50	18.96	19.69	23.14	32.50	42.02	36.28

*Note*: This analysis is based on 16,658 words present in the Educator’s Word Frequency Guide (Zeno 1995) for Grades 1 to 8

Obstruent consonant sounds in this analysis represented by *B*,* C*,* D*,* F*,* G*,* H*,* J*,* K*,* P*,* S*,* T*,* V*,* X*,* Z*

Sonorant consonant sounds in this analysis are represented by *M*,* N*,* L*

With the exception of the *n* rows as designated, all numbers are percentages (% omitted)

To answer RQ2, we employed a person-explanatory item-response model (De Boeck et al., 2017), a general linear mixed model (GLMM) in which random and fixed effects for persons and items are included. The model was estimated for a dichotomous outcome (use of a long-vowel pronunciation = 1; giving any other pronunciation = 0) using the *glmer* function in the *R* library *lme4* (Bates et al., 2015). The predictors in the GLMMs were grade (Grade 3 as reference; RQ2A), RD status (non-RD as reference; RQ2A), and vowel type (*E* as reference; RQ2B).

## Results

### RQ1: Are readers sensitive to the distribution of short and long vowel pronunciations in printed words?

For RQ1A, the pronunciations produced by the students generally showed extreme divergence from the distribution of long-vowel pronunciations in the lexical database. Table 4 shows the percentage of pronunciations by category in the database; Table 5 shows the percentage of pronunciations by category for the students in the study; Table 6 shows the students’ pronunciations by word; and Table 7 shows the χ² values and significance levels for the database-student pronunciation comparisons. There were some cases where the divergence was less extreme or non-significant. For 4 of the 8 categories for monosyllabic words (obstruent and sonorant *A* pronunciations, sonorant *I* pronunciations, and sonorant *O* pronunciations), there was not a significant χ² value, suggesting that the distribution of values did not differ significantly from student pronunciations and database values. One consistent pattern was the use of reduced-vowel pronunciations for the letter *E*.

**Table 5:** Percentage of long and short pronunciations for readers by group and grade

Letter	Vowel Type	All Readers	Typical Achievement (TA) Group (*n* = 60)				Reading Difficulty (RD) Group (*n* = 15)		
			All in TA Group	2nd Graders (*n* = 18)	3rd Graders (*n* = 19)	4th Graders (*n* = 23)	All in RD Group	3rd Graders (*n* = 9)	4th Graders (*n* = 6)
A									
	Short/æ/	70.1	56.7	67.2	78.9	70.4	50.7	53.3	73.3
	Long/eɪ/	12.4	12.5	12.2	11.1	13.9	12.0	16.7	12.0
E									
	Short/ɛ/	70.1	55.2	73.9	72.1	69.1	62.0	50.0	80.0
	Long/iː/	12.9	11.5	06.1	17.4	14.3	13.3	18.9	05.0
I									
	Short/ɪ/	78.9	80.7	76.9	82.0	82.0	71.7	62.1	86.1
	Long/aɪ/	07.8	07.8	06.1	07.9	09.1	07.8	09.3	05.6
O									
	Short/ɑ/or/ɔ/	41.1	39.7	54.6	31.6	34.8	46.7	38.9	58.3
	Long/oʊ/	44.6	47.9	25.0	61.8	54.3	31.1	28.7	34.7
All									
	Short	64.5	65.5	67.9	77.2	63.7	60.3	51.0	74.2
	Long	20.0	20.9	12.6	25.5	23.7	16.4	18.4	13.3

*K =* 32. All data are percentages (% omitted)

**Table 6:** Vowel pronunciation percentages by item

Item	Long	Short	Other		Item	Long	Short	Other
danal	5.2	79.2	15.6		pagal	17.3	60.0	22.7
dapic	2.7	81.3	0.2		panon*	10.7	77.3	12.0
davon	30.7	46.7	22.7		pasel	14.7	78.7	13.3
degid	25.3	48.0	26.7		pegel	6.7	81.3	12.0
delic	8.0	65.3	26.7		pelel	10.7	76.0	13.3
demal	13.3	69.3	17.3		pemel	9.3	78.7	13.0
dibal	6.7	81.3	12.0		pibel	12.0	66.7	21.3
ditel	9.3	77.3	13.3		pipal	14.7	74.7	10.6
divon	9.3	76.0	14.7		pivel	6.7	8.7	6.7
dolon	34.7	50.7	14.7		pobel	50.7	36.0	13.3
domal	45.3	34.7	20.0		polal	68.0	25.3	6.7
dosic	25.3	53.3	21.3		posel	42.7	50.7	6.6
lacal	9.3	60.0	30.7		tanen*	9.3	68.0	22.7
lagel	20.0	52.0	28.0		tatid	2.7	85.3	12.0
lanid	5.3	84.0	10.7		tavic	2.7	86.7	10.7
legic*	5.3	81.3	13.3		tegon	26.7	49.3	24.0
lelal	26.7	53.3	20.0		telit	5.73	81.3	13.3
lenic	2.7	88.0	9.3		telon	1.3	89.3	9.3
libon	2.7	84.0	13.3		tibic	2.7	85.3	12.0
licon	13.3	74.7	12.0		tigal	9.3	76.0	14.7
He ligic	2.7	76.0	21.3		tilic	4.0	88.0	8.0
locon	52.0	29.3	18.7		togal	57.3	28.0	14.7
lolic	33.3	61.3	5.3		tolel	58.7	16.0	25.3
losal	33.3	52.0	14.7		toson	33.3	56.0	10.7

All data are percentages (% omitted). *These words were not included in the final analysis because they begin with real words (e.g., *leg* in *legic*). Readers might have used real-word knowledge rather than probabilistic patterns to read these

**Table 7:** χ² Values comparing distribution of database vowel pronunciations vs. student pronunciations

Syllable (Syll) Pattern	A		E		I		O	
	Obstruent	Sonorant	Obstruent	Sonorant	Obstruent	Sonorant	Obstruent	Sonorant
Monosyll V_Only_	425.99	6.11*	36.64***	38.00***	47.11***	0.04	4230.40***	26.43***
Cramér’s *V*	0.832	0.199	0.398	0.248	0.517	0.016	1.14	0.185
Bisyll V_1_CV_2_	83.03***	111.04***	41.80***	1104.66***	451.13***	145.24**	48.17***	3.310
Cramér’s *V*	0.260	0.600	0.301	0.947	0.516	0.971	0.211	0.066
Polysyll V_a_CV_b_	228.69***	125.02***	67.37***	481.43***	196.40***	106.13***	92.41***	222.89***
Cramér’s *V*	0.431	0.637	0.382	0.625	0.340	0.340	0.293	0.538
V_Only_ and V_a_CV_b_	474.21***	123.94***	344.11***	479.19	280.16***	105.46***	147.24***	217.98***
Cramér’s *V*	0.620	0.634	0.863	0.624	0.407	0.828	0.370	0.532

Cramér’s *V* effect size interpretation: < 0.10 = negligible; > 0.10, ≤ 0.30 = small; > 0.30, ≤ 0.50 = medium; > 0.50 = large

Despite the large number of contrasts (*n* = 32), the fact that only 13% of all comparisons and 50% of monosyllabic comparisons were non-significant suggests these null findings are not due to Type I error. For RQ1B, most differences were statistically significant (Table 8), suggesting that students did not generally exhibit sensitivity to the fact that pronunciations of V_1_ in bisyllabic words in the lexicon vary depending on the subsequent consonant.

**Table 8:** Counts of bisyllabic words with vc patterns in database, pronunciation type distributions, and χ² comparison with student data

	Long		Other		Reduced		Short			
	*n*	%	*n*	%	*n*	%	*n*	%	*N*	χ²
ac	33	73.33	7	15.56	1	2.22	4	8.89	45	219.52***
ag	11	40.74	2	7.41	6	22.22	8	29.63	27	10.38**
an	1	2.94	3	8.82	13	38.24	17	50.00	34	35.42***
ap	9	45.00	4	20.00	4	20.00	3	15.00	20	106.92***
as	15	60.00	3	12.00	6	24.00	1	4.00	25	79.98***
at	41	49.40	17	20.48	6	7.23	19	22.89	83	319.42***
av	21	43.75	8	16.67	7	14.58	12	25.00	48	40.20***
e.g.	8	38.10	0	0.00	9	42.86	4	19.05	21	9.99**
el	6	14.63	0	0.00	31	75.61	4	9.76	41	98.78***
em	4	12.12	0	0.00	20	60.61	9	27.27	33	51.49***
en	9	25.00	1	2.78	14	38.89	12	33.33	36	30.47***
ib	4	66.67	0	0.00	0	0.00	2	33.33	6	–
ic	14	77.78	3	16.67	0	0.00	1	5.56	18	62.05***
ig	3	20.00	1	6.67	0	0.00	11	73.33	15	6.65*
il	17	73.91	0	0.00	0	0.00	6	26.09	23	
ip	5	83.33	0	0.00	0	0.00	1	16.67	6	–
it	12	52.17	4	17.39	0	0.00	7	30.43	23	55.59***
iv	14	40.00	0	0.00	2	5.71	19	54.29	35	30.32***
ob	12	66.67	0	0.00	0	0.00	6	33.33	18	24.64***
oc	13	68.42	0	0.00	4	21.05	2	10.53	19	1.87
og	4	57.14	0	0.00	0	0.00	3	42.86	7	–
ol	13	34.21	6	15.79	5	13.16	14	36.84	38	2.47
om	13	39.39	8	24.24	4	12.12	8	24.24	33	1.31
os	18	69.23	5	19.23	1	3.85	2	7.69	26	14.78**
Total	300		72		133		175		680	

### RQ2: Does sensitivity vary by student characteristics?

For our question concerning grade and reading ability status (RQ2A), the person-explanatory item-response models indicated a significant difference between grades and by RD status. From the student model (Table 9), the probability that a Grade 3 student (the reference group) used a long-vowel pronunciation was 16.7%, OR = 0.20, *p* <.001. Comparatively, the probability a Grade 2 student used a long-vowel pronunciation was just 3.8%, OR = 0.20, *p* <.001, indicating 0.20 times greater odds of using a long-vowel pronunciation than a Grade 3 student. A Grade 4 student’s probability of using a long-vowel pronunciation was not significantly different from that for a Grade 3 student. Further, controlling for grade, a student with RD was 57% less likely (OR = 0.43, *p* =.02) to use a long-vowel pronunciation than a peer without RD.

**Table 9:** RQ2 intercept and student models

Predictors	Intercept Model				Student Model			
	Odds Ratio	SE	*p*	95%CI	Odds Ratio	SE	*p*	95%CI
(Intercept)	**0.10**	**0.03**	**< 0.001**	**[0.06**,** 0.18]**	**0.20**	**0.07**	**< 0.001**	**[0.11**,** 0.39]**
Grade 2					**0.20**	**0.08**	**< 0.001**	**[0.10**,** 0.42]**
Grade 4					0.75	0.23	0.35	[0.42, 1.36]
RD					**0.43**	**0.15**	**0.02**	**[0.22**,** 0.86]**
**Random effects**								
𝜎^2^	3.29				3.29			
𝜏_id_	1.41				1.07			
𝜏_word_	2.28				2.27			
**Goodness of fit**								
Observations	3300				3300			
Model R^2^	0.00				0.06			
Conditional R^2^	0.53				0.53			
Deviance	2461.38				2442.20			
Δ𝜒^2^					**19.18**		**< 0.001**	
Δdf					3			
AIC	2467.38				2454.20			

Bold signifies significance at 𝛼 = 0.05; All ∆𝜒^2^ are comparisons to the intercept model

**Fig. 1:**
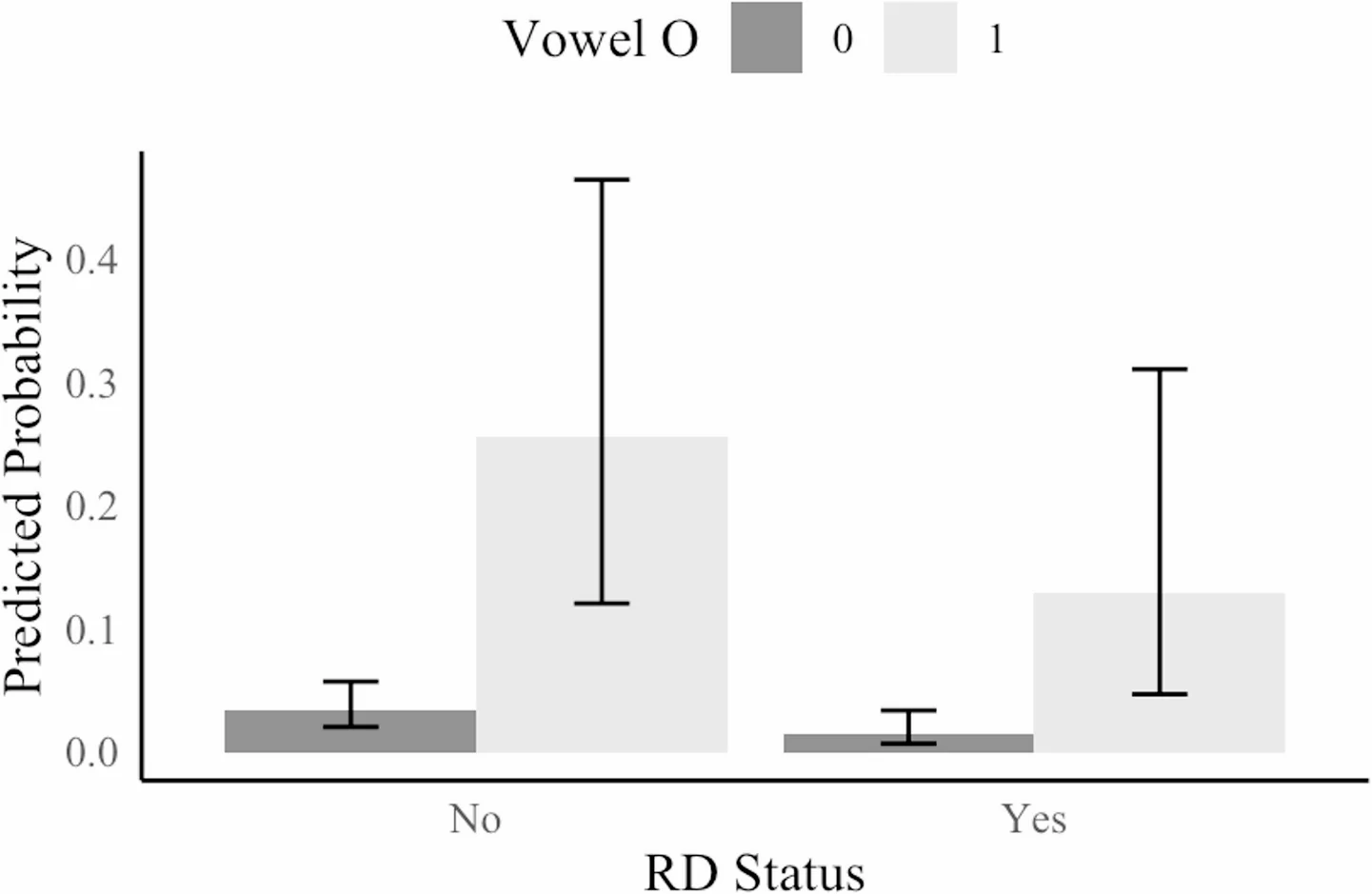
Predicted probability of long vowel pronunciation by RD status and vowel O

For our question concerning sensitivity differences by vowel letter (RQ2B), there was a significant difference in sensitivity across V_1_ vowels (see Table 10). The probability of producing a long-vowel pronunciation for *E* was 0.07, β = −2.61, *p* <.001. The probability for A was 0.07, β = −0.29, *p* =.49. The probability for *I* was 0.04, β = −0.60, *p* =.14, not significantly different than *E*. However, the probability of a long-vowel pronunciation when V_1_ was *O* significantly differed from *E* at 0.42, β = 2.28, *p* <.001. There was also an interaction between RD status and whether the pseudoword contained the letter *O*. For *A*,* E*, and *I*, the probability of a long-vowel pronunciation was generally low regardless of RD status. However, for the letter *O*, the probability of a long-vowel response was greater for students with and without RD than for other vowels but that this probability increase was larger for students without RD than those with it (see Fig. 1).

**Table 10:** RQ2 student and item and interaction models

Predictors	Student and Item Model				Interaction Model			
	Odds Ratio	SE	*p*	95%CI	Odds Ratio	SE	*p*	95%CI
(Intercept)	**0.14**	**0.05**	**< 0.001**	**[0.07**,** 0.30]**	**0.13**	**0.05**	**< 0.001**	**[0.07**,** 0.27]**
Grade								
Grade 2	**0.20**	**0.07**	**< 0.001**	**[0.10**,** 0.42]**	**0.19**	**0.07**	**0.01**	**[0.10**,** 0.41]**
Grade 4	0.75	0.23	0.34	[0.41, 1.36]	0.75	0.23	0.88	[0.41, 1.37]
RD Status								
RD Identified	**0.43**	**0.15**	**0.02**	**[0.22**,** 0.86]**	0.72	0.27	0.92	[0.21, 1.49]
Vowel Letter^a^								
*A*	0.75	0.32	0.49	[0.33, 1.71]	0.75	0.32	0.48	[0.33, 1.71]
*I*	0.55	0.22	0.14	[0.25, 1.22]	0.55	0.22	0.12	[0.25, 1.22]
*O*	**9.75**	**3.82**	**< 0.001**	**[4.53**,** 21.01]**	**11.88**	**4.72**	**< 0.001**	**[4.47**,** 25**,**90]**
Interaction								
RD Status × Vowel *O*					**0.37**	**0.10**	**< 0.001**	**[0.08**,** 0.63]**
**Random effects**								
𝜎^2^	3.29				3.29			
𝜏_id_	1.07				1.08			
𝜏_word_	0.69				0.70			
**Goodness of fit**								
Observations	3300				2951			
Model R^2^	0.26				0.26			
Conditional R^2^	0.52				0.52			
Deviance	2399.39				2386.92			
Δ𝜒^2^	**61.99**		**< 0.001**		**74.54**		**< 0.001**	
Δdf	6				7			
AIC	2417.39				2386.9			

Bold signifies significance at 𝛼 = 0.05; All ∆𝜒^2^ are comparisons to the intercept model

^a^ The letter *E* is the reference category

## Discussion

We conducted two analyses to determine whether students show sensitivity to the fact that some bisyllabic words are more likely to have long-vowel sounds than others. RQ1A results suggested students used long-vowel pronunciations at a different rate than predicted based on pronunciations of polysyllabic words in a large lexical database, but at a similar rate to monosyllabic words in 4 of 8 categories. When considering the vowel and trailing consonant for bisyllabic V_1_CV_2_words (RQ1B), students tended to use long vowel pronunciations at a different rate than predicted from the pronunciation distributions in the (Zeno 1995) database. The difference in distribution for bisyllabic words was also mostly true when accounting for the trailing consonant.

Additionally, we found different patterns across grade levels (RQ2A), indicating that younger students and students with RD seemed to pronounce single-letter vowels as they would experience them in monosyllabic words. We also found (RQ2B) students tended to use short sounds for V_1_ in bisyllabic for the letters *A*,* E*, and *I* more often that students did for *O*, where the long sound was used more often. Moreover, the difference in frequency of long-vowel use for *O* versus other letters was greater for students without RD than students with RD.

### Sensitivity to complex and subtle lexical patterns

We did not observe use of lexical patterns at frequencies approximating those in the bisyllabic words contained in texts they read daily. They much more closely approximate the distribution of pronunciation frequencies in monosyllabic words. In the connectionist framework, this is explained partly by the subtlety of the patterns and the likelihood that the reading system makes improvements earlier in acquisition that apply to more words, especially words that have more rule-like behavior. Rules may not exist, but consistency will allow the cognitive system to acquire some patterns quickly, as occurs with early readers in languages with transparent orthographies (Seymour et al. 2003). Specific to the type of word in the current project, subtlety, infrequency, and inconsistency of sound-spelling patterns all likely contributed to a prolonged period of learning to pronounce V_1_ in polysyllabic words correctly.

That said, the data also show some sensitivity to the greater likelihood of long-vowel pronunciations for polysyllabic words than for monosyllabic words. Were readers only using long-vowel pronunciations with the same frequency as monosyllabic words in the lexical database, they would almost never use long-vowel pronunciations. That was not what we observed. A particularly salient example of possible generalization comes from the sonorant *O* pronunciations. Cramér’s *V* was 0.066 for bisyllabic words and 0.185 for monosyllabic words, indicating more frequent use of long-vowel pronunciations for V_1_ than expected based on the long-vowel *O* frequencies for monosyllabic words (last two columns of Table 7). This reinforces the idea of gradual learning we would expect to further emerge with experience.

Looking across all the values reported in Table 7, the magnitude of Cramér’s *V* varied considerably by category. For example, the values for bisyllabic words were larger than those for all polysyllabic words in any position (notably, sonorant *E* and obstruent *I*). These differences suggest that learners develop sensitivity not only to the patterns as they occur in shorter words but to their experience with words of various lengths. The complexity introduced by additional inputs and outputs does not necessarily attenuate the influence of experiences reading these words despite that longer words require representation of more inputs and output features.

The evidence for influence of context is not strongly supported in our analysis of pronunciations of V_1_ when accounting for the presence of particular C_1_ letters (e.g., *ac* as in *lacal*). Table 8 provides the χ² values for the comparison between long pronunciations of vowels given the trailing consonant in the lexical database and student long-vowel use. Their magnitude does not strongly suggest an influence of this immediate context, although the magnitude varies, and the χ² statistics for *oc*, *ol*, and *om* are not significant. Taken together with the bisyllabic and all polysyllabic results, questions remain about the influence of various lexical factors in reading acquisition for elementary-age readers. Experiments isolating and testing the influence of features like word length would advance the field’s understanding of salient factors in acquisition of word recognition skills.

### Variability by grade and reading ability

There are clear patterns based on age and reading ability—with younger readers and those with reading difficulty using short-vowel pronunciations more often. This result aligns with our hypothesis. It raises the question of the locus of the problem. One possibility concerns reading experience. Younger readers with typical achievement necessarily have less experience than older readers. Students with reading difficulty generally have less experience than their peers with typical achievement because the difficulty makes reading slower (fewer words encountered per minute than their peers) and undesirable (low likelihood of electing to read).

The other possibility involves cognitive factors. Sawi & Rueckl (2019) highlighted developmental differences in memory (explicit or implicit, declarative or procedural) that may affect the way that learners process sensory information. In other words, younger readers profit less from experience reading than their older peers because they perceive, encode, and store linguistic information more slowly and perhaps relying on different memory systems than older students. Reading difficulty may involve the same speed and memory differences as younger students. There are other possibilities including the strength of the phonological system, learning rate, and learning capacity (Harm & Seidenberg, 1999), among others. This study does not address these issues, but but the grade- and RD-related variability we observed suggests that further exploration of experiential and cognitive factors in the acquisition of complex lexical information is warranted.

### Contextualizing the computational account

The present study provides evidence that supports the parallel distributed processing triangle model Given the quasiregularity of English, few features are almost entirely consistent, and the pronunciation of single-letter vowels in polysyllabic words provides a salient example of this inconsistency. However, the pronunciations are not arbitrary. In V_1_CCV_2_ words, V_1_ is generally short; thus, a double-consonant-short-vowel rule is useful. In V_1_CV_2_ words, there is still a tendency toward a single-consonant-long-vowel pronunciation relative to the mostly-short-vowel pronunciations for monosyllabic words—but the tendency cannot be explained entirely—or even mostly—by a set of rules—although there have been many attempts to do so (see Kearns et al., for examples). As Coltheart & Rastle 2000 observed, even a single factor involved in polysyllabic word reading (stress) requires an extensive set of rules that learners would be unlikely to apply. In short, the results strongly align with the parallel distributed processing model designed to accommodate pattern learning through adaptation of the network via experience rather than acquisition of specific rules employed in word recognition.

Although we interpret these results as broadly supportive of a computational account, (Treiman & Kessler 2025) considered other explanations why the statistical patterns in the language and the pronunciations given by children and adults are not aligned. They considered that nuanced and relatively infrequent letter-sound dependencies are less likely to affect readers than more apparent ones. Even in a statistical model, the learner may not observe these subtle patterns. Another explanation is that the reader often need not try a more complex pattern if a simpler one is adequate to access the phonological representation in the lexicon (satisficing; see also (Perry et al. 2010). Research on *set for variability* aligns with this finding (Kearns et al. 2016; Edwards et al. 2022; Steacy et al. 2023); researchers have observed that children with greater skill in linking incorrectly pronounced words to the correct spoken word have overall better reading skills. In the current case, it might not be necessary to acquire sensitivity to an abstruse letter-sound association if the pronunciation of a given word can be retrieved using the other letters. For example, if readers use only short pronunciations for vowel letters in polysyllabic words, the consonants provide enough information to locate the correct entry in the lexicon. Satisficing therefore explains performance. In the present study, these pseudowords have no connection with a lexical entry, so the tendency to use short vowels--but some use of long vowels--must be explained by some mechanism that involves sensitivity to patterns without any direct teaching.

These possibilities raise interesting questions about the nature of statistical learning, especially with regard to the frequency of the patterns readers observe. In tests of the triangle model, models develop sensitivity to statistical patterns like the divergent pronunciations of *ea* with *d *or without (e.g., *seat* vs. *bread*; Plaut et al. 1996), but most have not explored effects in polysyllabic words (but see Perry et al. 2010). Longer words necessarily require use of more cognitive resources, raising the possibility that learners acquire sensitivity to these patterns more slowly because the pattern of activation is distributed over a more diffuse network. (e.g., (Harm & Seidenberg 2004).

A final related point concerns overexposure to high-frequency patterns. Networks settle into states that accommodate both *was* and *gas*, but it remains unclear how exposure to *was* changes the network. It is possible that the constant input of high-frequency words with short vowels biases the network toward these pronunciations long after they were first acquired. This is another largely unexplored and interesting area of computational research.

### Implications for practice

Given that these data are correlational and cross-sectional, instructional recommendations would not be appropriate. However, the alignment with the triangle model suggests that teachers should carefully consider the extent to which they provide instruction on putative—but subtle, abstruse, and infrequent—features of the language. Many educators have tried to make reading easier for children by providing them with a set of rules they can apply to make the pronunciation of unfamiliar words easier. However, the quasiregularity of English leads necessarily to the identification of rules that apply to very few words (e.g., *eigh* for /e/ in *neighbor*). Should children learn the (putative) *eigh*, *ea*,* ei*, and *ey* pronunciations of /e/ in addition to *a*,* a-e*, a.i.,* and ay*? Educators today would debate this question with great fervor and no consensus. A given educator's answer depends on their theory of learning: Either (a) readers need a robust set of rules learners can apply or (b) experience is adequate to acquire knowledge of the lexicon and few explicitly stated rules are needed. Rules have diminishing returns, but the data in this paper suggest that learners can develop sensitivity to subtle patterns. The question is whether the benefit of explicating these lexical tendencies outweighs the cognitive demand involved in storing and retrieving this pattern information applicable to a limited number of words. This study raises the possibility that instruction on the apparent syllable structures observed in English may not provide an adequate benefit to justify the time spent modeling, practicing, and mastering the ability to recognize infrequent sound-spelling associations.

### Limitations

There are several limitations to our findings due to method. A particularly important one is we did not compare performance on V_1_CV_2_ words with V_1_CCV_2_ bisyllabic words as (Treiman et al. 2023) did with adult readers. Our argument that early readers’ pronunciations of vowels do not reflect their distribution in the language must be tempered because we have no data concerning other words. Given previous findings with adults, we would expect that early readers would use a short-vowel pronunciation more often in V_1_CCV_2_ bisyllabic words. Even so, our data are incomplete without examining these pronunciations. A similar concern could be raised about the absence of longer polysyllabic words.

Second, the sample of students does not represent the population of students in Grade 2–4. The focus on typical achievers and students with RD allowed us to answer RQ2, but this created gaps in the distribution of students that limits the generalizability of the results. Moreover, the grade-based comparisons were based only on cross-sectional data. Grade-based performance differed, but it is possible that this was due in part to characteristics of students in each grade; longitudinal data would be more convincing. Additional cross-sectional data for a less constrained distribution of students across more grades would also reinforce these results.

We also have no information about students’ instructional history, except whether they participated in reading intervention: Most children with RD did, but we did not ask specifically whether they learned strategies for identifying and applying the putative pattern of long-vowel pronunciation for V_1_ in V_1_CV_2_ words. This is an obvious limitation, but the results run counter to the idea that instruction for leads to overgeneralization (i.e., greater use of more long-vowel pronunciations than would be expected based on the lexical statistics). If students had learned such a strategy, the impact does not appear to have suppressed the influence of prior experience with other pronunciations.

Fourth, the sample size was small, which could raise concerns about statistical power, particularly given that we interpret some null findings as theoretically meaningful (i.e., student performance matching monosyllabic patterns in the word database). Effect size analyses (Cramér’s *V*; see Table 7) help address this concern. The non-significant comparisons in which students appeared to follow monosyllabic patterns showed negligible effect sizes (V < 0.10 or < 0.30), suggesting these were not Type II errors but rather genuine similarities to database frequencies. However, Cramér’s *V* cannot rule out the possibility that null effects were the result of low power or Type II errors, particularly in small subgroups like our RD sample (*n* = 15).

Last, the analyses could have been expanded to explore other distributional differences. For example, we only examined whether student pronunciations for V_1_ varied based on the subsequent consonant for bisyllabic words. We chose not to include any frequency-weighted analyses for RQ1. These decisions were based on the concern that additional analyses would increase Type I error and that these were not part of our planned contrasts because we thought the current set of analyses was best for answering these questions. Despite these limitations, this study contributes to our understanding of how statistical learning mechanisms support early decoding. Future research should examine instructional interventions that scaffold early readers’ sensitivity to subtle vowel-consonant patterns in polysyllabic words.

## Conclusion

The results of this study contribute to our understanding of elementary-age readers’ sensitivity to subtle sound-spelling patterns in polysyllabic words. We believe these results indicate that statistical learning is a powerful mechanism in reading acquisition but happens gradually and may be slowed by reading difficulty. These results should also give educators pause before engaging in extensive instruction on infrequent sound-spelling associations despite their laudable and understandable intention to structure the learning environment to maximize the speed of reading acquisition, because the benefit is unknown but not necessarily helpful for improving word recognition skills.

## Footnotes

1 Multi-letter graphemes are also used to represent these sounds, especially long ones (e.g., *ea*,* ee*, and *ey* for/i/).

2 This notation will be used throughout to identify the type of letter (vowel [V] or consonant [C]). Subscripts will be used to notate the vowel’s position in the word. The first vowel (V_1_) of the targets (V_1_CV_2_) used in this study were of particular interest.

3 The written *sk* in *basket* encodes the consonant cluster in the phonological form; the *BB* in *rabbit* is an orthographic convention used in cases that there is only one consonant phoneme between the vowels.

4 We use the term *word reading* to describe the activation of the correct pronunciation and semantic features for a given written word.
